# Patient-reported experience in device-assisted enteroscopy: Conscious sedation vs. general anesthesia

**DOI:** 10.1055/a-2870-7457

**Published:** 2026-06-08

**Authors:** Fintan John O'Hara, Edric Leung, Conor Costigan, Deirdre McNamara

**Affiliations:** 1 Department of Gastroenterology 57976 Tallaght University HospitalDublinLeinsterIreland; 2 Trinity Academic Gastroenterology GroupDepartment of Medicine 8809 Trinity College DublinDublinIreland

**Keywords:** Endoscopy Small Bowel, Small bowel endoscopy, Quality and logistical aspects, Sedation and monitoring, Quality management, Performance and complications

## Abstract

**Background and study aims:**

Device-assisted enteroscopy (DAE) plays a critical role in evaluation and treatment of small bowel disorders, with double-balloon enteroscopy (DBE) being the most widely used technique. Sedation strategies for enteroscopy remain a topic of debate, with conscious sedation (CS) and general anesthesia (GA) both being utilized. Our aim was to assess patient-reported experience measures (PREMs) for DAE, comparing CS and GA to determine their impact on patient satisfaction, procedure success, and pain perception.

**Patients and methods:**

PREMs were prospectively collected from 118 unselected patients referred to a single center for DAE between July 2022 and March 2025. PREMs, procedure details, and sedation techniques were analyzed.

**Results:**

Overall patient satisfaction was good with 100% being satisfied or very satisfied. A significantly higher proportion in the GA group reporting being "very satisfied, (92.3% GA vs 71.0% CS, *P* = 0.004). Despite experiencing greater discomfort, albeit within acceptable parameters (median 0, average 1.9) the CS group still reported high overall satisfaction. Technical success was achieved in 98.3% of cases and there was no statistical difference in technical outcomes between the groups.

**Conclusions:**

Although GA provides optimal conditions for enteroscopy, in our experience, CS remains an effective and acceptable option for patients, maintaining accessibility to the procedure. Our study supports use of PREMs in enteroscopy as a quality assurance measure and underscores the need for individualized sedation strategies to optimize patient care.

## Introduction

Device-assisted enteroscopy (DAE) was introduced in 2001 and has since revolutionized investigation and treatment of small bowel disorders. DAE allows for deep examination beyond the reach of push enteroscopy, facilitating biopsy, tattoo placement, and treatment of bleeding lesions 1 . Since it was first described by Yamamoto et al. over two decades ago, the repertoire of DAE techniques has expanded to include double-balloon enteroscopy (DBE) and single-balloon enteroscopy (SBE) 2 3 . DAE is now well established as the procedure of choice for patients requiring further endoscopic evaluation, biopsy, or endotherapy of a variety of small bowel pathologies. The procedure remains time-intensive, with mean anterograde and retrograde durations of 70 and 85 minutes, respectively, in published series. Major complications occur in 0.72% of diagnostic cases, increasing to 4.3% when therapeutic intervention is involved.

Key performance indicators have been established to improve and standardize quality of care across endoscopic procedures 4 5 . In 2019, the European Society of Gastrointestinal Endoscopy (ESGE) proposed a set of quality performance measures for small bowel endoscopy which has been updated recently 5 6 . Although these provide important benchmarks for DAE services, they predominantly focus on technical outcomes, diagnostic yield, and adverse events (AEs), with comparatively limited emphasis on the patient experience.

Sedation choice in enteroscopy remains an area of ongoing debate 7 8 . Adequate sedation is essential to optimize procedure success and patient tolerance. Sedation practices vary by region and institutional guidelines. The British Society of Gastroenterology recommends a risk-stratified approach, advocating conscious sedation for diagnostic procedures and deeper sedation for therapeutic interventions 9 . The ESGE favors propofol-based sedation for improved patient comfort and procedure efficiency 5 , whereas American guidelines support anesthesia-led sedation in complex or prolonged cases. However, these recommendations are largely informed by technical- and clinician-reported outcomes rather than direct patient-reported experience.

Large-scale data evaluating performance measures in DAE remain limited. The recently published DEEP-UK study assessed national performance metrics and demonstrated that UK practice met ESGE quality benchmarks, with high diagnostic and therapeutic yields and low major AE rates. However, variation in procedure volume and its potential impact on performance and optimization of sedation strategies were highlighted 9 . Notably, patient-reported experience was not evaluated in this national analysis.

There has been one published study that investigated tolerability of DBE when compared with esophagogastroduodenoscopy (EGD) and colonoscopy 10 . Although tolerability of retrograde DBE was comparable to colonoscopy, more than half of patients undergoing antegrade DBE did not achieve satisfactory sedation and reported poorer tolerability than during EGD. Tolerability in this and similar studies has primarily been assessed using clinician- or nurse-reported measures rather than direct patient reporting.

Although there is no standard method for assessing patient comfort in endoscopy, several scores exist, such as the La Crosse intra-endoscopy sedation comfort score 11 and the nurse-assessed patient comfort score (NAPCOMS) 12 . The commonly used Gloucester score 13 has recently been shown to underestimate pain compared with a patient-reported measure in colonoscopy 14 . Patient-reported outcomes are increasingly recognized as essential components of quality assessment in endoscopy and are central to delivering patient-centered care 15 .

Despite this growing emphasis on patient-centered metrics, a clear evidence gap remains in DAE. Comparative patient-reported experience data are scarce and no prospective real-world studies have systematically evaluated the impact of sedation modality on patient experience during DAE. Furthermore, although deeper levels of sedation may facilitate procedure completion and therapeutic intervention, the balance between maximizing procedure depth and maintaining patient comfort has not been clearly defined. To date, no published data have specifically reported patient-reported experience measures (PREMs) in DAE using a validated instrument.

ENDOPREM is a validated tool for assessment of patient-reported outcomes in endoscopy, designed to assess patient satisfaction, comfort, communication, and overall experience 16 . It provides standardized feedback to improve endoscopy services, enhance patient-centered care, and identify areas for quality improvement in gastrointestinal endoscopy procedures.

The aim of this study was to assess patient-reported tolerability and experience of DAE using the validated ENDOPREM tool and to compare patient experience and procedure success between procedures performed under conscious sedation (CS) and general anesthesia (GA).

## Patients and methods

Our high-volume enteroscopy service (310 procedures in 2024) provides DAE under CS in the endoscopy unit as well as under general an anesthetic in our offsite day surgery center. Procedures are performed by two experienced endoscopists, both performing DAE with CS and GA in an unselected fashion. A combination of fentanyl and midazolam +/- hyoscine butyl bromide, administered by the endoscopist, was used for CS cases, whereas consultant anesthetists provided GA for the GA cases. Propofol sedation was not included because it is not routinely available in our unit.

We performed a prospective observational cohort study of unselected DAE patients treated in both settings between July 2022 and March 2025. Patients were asked to complete a modified ENDOPREM survey post-procedure prior to discharge on the day of the procedure to maximize participation. The ENDOPREM questionnaire was modified to include enteroscopy, both antegrade and retrograde, as a procedure type, but the rest of the survey remained unchanged. There was no alteration to standard clinical care, sedation strategy, or procedure decision-making.

This study was approved as a quality improvement initiative by the institutional Research and Ethics Committee, which determined that formal written consent was not required. The study conformed to the principles of the Declaration of Helsinki. Patients received information about the survey and return of a completed questionnaire implied consent. All data were pseudonymized and managed in accordance with guidance from the Health Information and Quality Authority, Ireland’s national body for health information governance, and institutional data protection policies.

As well as the PREMs from the survey, pseudonymized data including demographics, procedure indications, technical success, sedation dosages, and procedure duration were collected. Post-procedure assessments measured pain, discomfort, and distress on a numerical rating scale (0–10). The primary endpoints were overall patient satisfaction, patient-reported intra-procedure and post-procedure pain scores and procedure technical success. Technical success was defined as successful intubation and advancement of the enteroscope to the intended anatomical target or achieving the predefined diagnostic or therapeutic goal without premature termination due to patient intolerance, sedation failure, or procedure complication. Secondary outcomes were complication rates, sedation dosages in CS cases, procedure duration, and diagnostic yield.

Statistical analysis was conducted using SPSS v20. Nonparametric tests (Mann–Whitney U) compared between CS and GA groups, with a significance threshold of *P* < 0.05.

## Results

A total of 118 patients completed the survey and were included, 66 (56%) CS and 52 (44%) GA DAE cases. In all 52 (44.1%) were female with a mean age of 52.2 years (range 21–84 years). Patients undergoing GA procedures were younger, 40.2 years vs 61.6 years ( *P* < 0.001). This is, in part, was due to the exclusion criteria for our day surgery center, where significant comorbidities excluded some patients from GA at the anesthetic preassessment stage. Overall, 94 (79.7%) were antegrade whereas 24 (20.3%) were retrograde procedures. CS patients were more likely to have had an antegrade enteroscopy (87.9% vs 69.2%, *P* = 0.02). DBE was the most common technique 114 (96.6%), with only four (3.4%) SBE included ( Table 1 ).

**Table:** **Table 1** Study population.

**Characteristic**	**Overall (n = 118)**	**Conscious sedation (n = 66)**	**General anesthesia (n = 52)**	***P* value **
Age, mean ± SD (years)	52.2 ± 18.9	61.7 ± 16.5	40.2 ± 14.1	< 0.001
Female sex, n (%)	52 (44.1%)	28 (53.8%)	24 (46.2%)	0.005
Male sex, n (%)	66 (55.9%)	38 (57.6%)	28 (42.4%)	0.012
**Enteroscopy route, n (%)**				0.017
Antegrade DAE (ADBE)	94 (79.7%)	58 (87.9%)	36 (69.2%)	
Retrograde DAE (RDBE)	24 (20.3%)	8 (12.1%)	16 (30.8%)	
ADBE, advanced double-balloon enteroscopy; DAE, device-assisted enteroscopy; RDBE, retrograde double-balloon enteroscopy; SD, standard deviation.				

### Procedure and technical data

In the CS group, median midazolam dose was 5 mg (range 2–10 mg). For fentanyl, median dose was 75 µg (range 25–100 µg). The mean dose of fentanyl was 78.2 µg. One patient in the CS group had an additional 25 mg of pethidine administered. Also, 39% of patients in the CS group received hyoscine butyl bromide with a median dose of 10 mg. Two patients (3.0%) in the CS group did not tolerate intubation but later successfully underwent the procedure under GA. Thus, technical success was achieved in 97% of the CS group (64/66) vs. 100% (52/52) in the GA group, with an overall technical success rate for the cohort of 98.3%.

Mean insertion depth for antegrade enteroscopy was 235 cm (interquartile range [IQR] 160–300), with total enteroscopy achieved in 0% of cases ( Table 2 ). There was a statistically significant difference in insertion depth between the two groups, with mean depth of insertion in CS of 258 cm (IQR 180–350 cm) vs. 199 cm for GA (IQR 138–210 cm) ( *P* = 0.0078).

**Table:** **Table 2** Depth of insertion during ADBE.

	**Conscious sedation**	**General anesthesia**	
n	58	36	
average depth (cm)	258	199	*P* = 0.008
IQR	170	73	
ADBE, antegrade double balloon enteroscopy; IQR, interquartile range.			

Median procedure duration for antegrade enteroscopy was 57 minutes (IQR 37–70) and was similar between both groups (58 min vs 56 min; *P* = 0.6) ( Table 3 ).

**Table:** **Table 3** Time for ADBE.

	**Conscious sedation**	**General anesthesia**	
n	58	36	
average time (mins)	58	56	*P* = 0.5953
IQR	19	42	
ADBE, antegrade double balloon enteroscopy; IQR, interquartile range.			

Indication for DAE differed between the two groups, with a significantly higher proportion of patients in the CS group undergoing the procedure for investigation of suspected/known small bowel bleeding (40/66 vs. 20/52, *P* < 0.001). This is expected because significant anemia is an exclusion criterion for GA in our anesthetic assessment ( Table 4 ). Overall diagnostic yield was high and was similar in the two groups (80.0% vs. 79.0%; *P* = 0.37). In keeping with the indication bias, vascular lesions were more common in the CS group (47% vs 10%, respectively). Although inflammatory lesions, ulcers, erosions, and strictures were more common findings in the GA group at 31% vs 12%. Table 5 . Therapeutic interventions were performed in 51 procedures (43%) and included argon plasma coagulation, endoscopic clipping, polypectomy, adrenaline injection, and stricture dilation. The overall therapeutic success rate was 96% (51/53), based on intention to treat and was similar in both groups.

**Table:** **Table 4** Indication for DAE by group.

**n = 118**	**Conscious sedation**	**General anesthesia**	
Anemia/SSBB	40	20	*P* = 0.001
Radiological abnormality	9	6	
Suspected Crohn’s disease	8	13	
Lesion on capsule	7	9	
Stricture dilatation	2	4	
DAE, device-assisted enteroscopy; SSBB, suspected small bowel bleeding.			

**Table:** **Table 5** Diagnostic yield by group.

**n = 118**	**Conscious sedation**		**General anesthesia**		
Normal	13	20%	11	21.0%	
Vascular lesion	31	47%	5	10%	*P* = 0.001
Inflammatory lesions	8	12%	16	31%	
Stricture	2	3%	6	12%	
Tumor	4	6%	4	8%	
Polyps	4	6%	8	15%	
Others	4	6%	2	4%	

### Adverse events

One episode of laryngospasm occurred in the GA cohort. This occurred at the end of a technically successful antegrade double balloon enteroscopy and was managed with airway repositioning and positive pressure ventilation, without the need for endotracheal intubation or procedure abandonment. The patient was admitted overnight for observation and recovered fully with no sequelae. Although laryngospasm is a recognized complication of GA, particularly in upper gastrointestinal procedures, its occurrence in this study did not result in prolonged hospitalization or adverse clinical outcome. There were no serious AEs in the CS group.

### Pain scores

Pain experienced during the procedure was graded from 0 (no discomfort) to 10 (worst discomfort imaginable). In general, DAE procedures were well tolerated in both groups. As expected, almost all GA patients reported no pain during the procedure. As such, there was a significant difference in patient-reported none or minimal discomfort (comfort scores 0–1) with use of GA versus CS (99.7% vs. 68.5%; *P* < 0.001). The CS group reported a median pain score of 0 (feeling no pain) with an average pain score of 1.9 (range 0–10). Although 10 patients (15.2%) in the CS group reported significant levels of discomfort (5 or greater), interestingly, all of these patients were satisfied (40%) or very satisfied (60%) with their test. Poor patient tolerance limited 6% of procedures (N = 2), both antegrade, performed under CS, compared with none in the GA group.

### Patient satisfaction

All participants in both the GA and CS groups (n = 118, 100%) who completed the survey reported overall satisfaction with their procedure, as either “satisfied” or “very satisfied”. A significantly higher degree of patients in the GA group were “very satisfied” with their experience (92.3% GA vs 71.0% CS, *P* = 0.004).

## Discussion

This study represents the largest prospective evaluation to date of PREMs in DAE, directly comparing CS and GA. Using a validated patient-reported tool 16 , we demonstrate that both sedation strategies are associated with high overall satisfaction and technical success, despite measurable differences in procedural discomfort.

Overall satisfaction was uniformly high, with all respondents reporting being either “satisfied” or “very satisfied.” Although a greater proportion of patients undergoing GA reported being “very satisfied,” patients in the CS group also reported high satisfaction, even among those experiencing moderate discomfort. This finding highlights an important distinction between discomfort and overall experience; procedure pain alone does not necessarily determine patient-perceived quality of care. Incorporation of a validated PREM provides a more nuanced assessment of tolerability than clinician-reported comfort scores, which have previously been shown to underestimate patient discomfort 14 .

Technical success was high in both groups (98.3% overall), with no statistically significant difference between sedation modalities. Although two CS procedures were limited by patient tolerance, overall diagnostic yield and therapeutic success were not adversely affected. This suggests that, in appropriately selected patients, CS does not materially compromise procedural effectiveness.

Interestingly, insertion depth was significantly lower in the GA cohort despite comparable technical success rates. This finding may appear counterintuitive because deeper sedation might be expected to facilitate prolonged advancement and therapeutic maneuvers. Several explanations may account for this observation. First, sedation allocation was not randomized, and patients selected for GA may have differed in procedure complexity, prior abdominal surgery, or anatomical factors limiting advancement. Second, under GA, absence of patient discomfort cues may alter operator pacing or threshold for continued advancement once the clinical target has been reached. Third, differences in patient positioning, abdominal wall tone, or insufflation dynamics under GA may subtly influence small bowel pleating and scope progression. Importantly, technical success and therapeutic yield were maintained, suggesting that reduced insertion depth did not compromise clinical outcomes. Given the non-randomized design and potential confounding factors, this finding should be interpreted cautiously and does not, in isolation, support modification of sedation strategy solely to maximize insertion depth.

Sedation strategy in DAE remains an area of ongoing debate. The recent update to ESGE performance measures for small bowel endoscopy 5 and the DEEP-UK study 9 have suggested consideration of deeper sedation for DAE. However, these recommendations are largely based on technical and clinician-reported metrics rather than direct patient experience. Our findings demonstrate that CS remains well tolerated in most patients, with median pain scores of 0 and low average discomfort. Even among patients reporting higher discomfort scores, satisfaction remained high.

Previous work comparing tolerability of DBE with other endoscopic procedures has suggested that DBE may be less well tolerated than standard upper or lower endoscopy under CS 8 17 . Despite this, in our cohort, discomfort levels were generally low and did not translate into reduced satisfaction or procedural success.

Exclusive reliance on GA or deep sedation may have implications for service delivery and access. In our practice, a substantial proportion of patients undergoing DAE for small bowel bleeding did not meet criteria for GA due to comorbidities or anesthetic risk. For such patients, CS provides a safe and accessible alternative. These findings support a tailored, patient-centered approach rather than a uniform sedation mandate.

Each sedation modality carries distinct risks and benefits. CS is widely available but may be associated with greater intra-procedural awareness and potential cardiorespiratory effects. GA provides optimal procedure conditions and eliminates discomfort but introduces risks related to airway management and hemodynamic instability. In large colonoscopy cohorts, use of anesthesia services has been associated with increased AEs 18 , although direct extrapolation to DAE must be cautious. In our cohort, AEs were rare. One patient in the GA group developed laryngospasm requiring overnight observation but recovered fully without sequelae. No serious procedure complications occurred in either group.

Propofol-based deep sedation has been associated with favorable patient comfort and satisfaction profiles compared with traditional benzodiazepine-opioid regimens in endoscopic procedures, including colonoscopy, and may be associated with shorter recovery times 17 18 . However, its availability and governance vary across healthcare systems. Propofol was not routinely utilized in our cohort, where GA rather than deep propofol sedation was employed when anesthetic support was required. Future prospective comparative studies evaluating propofol-based sedation alongside CS and GA in DAE would further inform optimal sedation strategy.

This study has several strengths. It prospectively evaluated consecutive patients in a high-volume DAE center and utilized a validated patient-reported instrument 16 , addressing a recognized gap in existing quality metrics for small bowel endoscopy 5 6 .

However, several limitations warrant consideration. First, this was a single-center study conducted in a high-volume unit, and outcomes may not be directly generalizable to lower-volume centers with different case-mix or sedation practices. Second, sedation allocation was not randomized and was influenced by anesthetic pre-assessment criteria, introducing potential selection bias between groups. Older/comorbid patients were more likely to receive CS, which may bias outcomes and affect comparability. There was also no propofol sedation arm, which should be looked at in further trials. Third, although the response rate of 62% compares favorably with similar endoscopy-based PREM studies, non-response bias cannot be excluded. Patients who did not complete the questionnaire may have had systematically different experiences, either more negative (e.g. greater discomfort or dissatisfaction) or more positive (e.g. rapid recovery and early discharge), which could influence the overall experience scores. If non-responders differed in clinical complexity, sedation modality, or procedure outcomes, which may also limit the generalizability of the findings. Finally, satisfaction-based measures may demonstrate a ceiling effect and interpretation, therefore, should be considered alongside objective pain scores.

Taken together, our findings suggest that both CS and GA are acceptable and effective sedation strategies for DAE when applied within an appropriate clinical framework. CS may be suitable for diagnostic antegrade procedures in carefully selected patients, particularly where GA access is limited or anesthetic risk is elevated. GA may be preferable in younger patients, retrograde procedures, or complex therapeutic cases where prolonged intervention is anticipated. Individualized sedation planning that integrates procedure complexity, patient comorbidity, institutional resources, and patient preference is likely to optimize both patient experience and service delivery.

## Conclusions

In this prospective evaluation of patient-reported experience in DAE, both CS and GA were associated with high levels of patient satisfaction and acceptable tolerability. Although GA was associated with comparable technical success and patient experience, insertion depth differences likely reflected case selection rather than sedation effect alone.

These findings support an individualized approach to sedation selection in DAE. CS may be appropriate for patients undergoing diagnostic procedures with anticipated lower complexity, particularly when minimizing resource utilization is a priority. GA may be considered for younger patients, those with inflammatory pathology or strictures, or when prolonged therapeutic intervention is anticipated.

Sedation decisions, therefore, should incorporate procedure indication, anticipated complexity, patient comorbidity, and patient preference, rather than aiming solely to maximize insertion depth. Further multicenter studies are warranted to refine sedation strategies in enteroscopy.

## Supplementary Material

Supplementary Material Zusatzmaterial
