# Supplementary material for: Patient-reported experience in device-assisted enteroscopy: Conscious sedation vs. general anesthesia
Source: Endosc Int Open. 2026 Jun 8;14:a28707457. doi: 10.1055/a-2870-7457 (PMC13289828; doi:10.1055/a-2870-7457)
Supplement: Supplementary file 1 — Supplementary Material [file 10-1055-a-2870-7457_28723330.pdf]

Thieme

Supplementary Material:

Fintan O'Hara, Edric Leung, Conor Costigan, Deirdre McNamara

Patient-reported experience in device-assisted enteroscopy: conscious sedation versus general anaesthesia

# Patient Questionnaire

## Patient Reported Experience Measure for Gastrointestinal Endoscopy

Endosc Int Open | DOI 10.1055/a-2870-7457 | © The Author(s).

Thank you for agreeing to take part in this survey - we really appreciate you taking the time to complete this questionnaire about your experience of having an endoscopy (camera) test or CT colonoscopy (scan).

## Completing the survey

Please fill in this questionnaire for your most recent test.

The survey will take about 10 minutes to complete. All of the questions are important so please try to answer them all. There are no right or wrong answers - we are just interested in hearing about your experience so we can make improvements to the way we deliver the service.

If you need help filling in the survey you can ask someone to help you.



Endosc Int Open | DOI 10.1055/a-2870-7457 | © The Author(s).

## Section A: Completing this survey

Please answer all of the questions in this section by writing in the boxes or putting a '✔' next to the answer that applies to you.

A1. Please fill in today's date d: ☐☐ m: ☐☐ y: ☐☐☐☐

A2. How long ago was your most recent test? Weeks:__________ Days: __________

A3. Please fill in your age (in years) ☐

A4. Are you? ❍ Male ❍ Female

A5. How many years of full time education you have completed? ☐

A6. Please tell us if someone is helping you complete this survey

I am completing this survey by myself ❍

Someone is helping me complete the survey ❍



Endosc Int Open | DOI 10.1055/a-2870-7457 | © The Author(s).

A8. Which test did you have on this occasion?

Antegrade Enteroscopy
*(Balloon camera inserted through the mouth)* ○

Retrograde Enteroscopy
*(Balloon camera inserted into back passage)* ○

Gastroscopy
*(Camera inserted through mouth into the stomach)* ○

Colonoscopy
*(Camera inserted into back passage* ○

If in doubt please ask

A9. Have you had another camera test in the past?

Yes ○ No ○

Excluding your most recent test, please indicate which tests and how many you have had

Colonoscopy
*(Camera or tube inserted through the back passage)* ○ Number ____

Gastroscopy
*(Camera or tube inserted through the mouth into the stomach)* ○ Number ____

Antegrade Enteroscopy
*(Balloon camera inserted through the mouth)* ○ Number ____

Retrograde Enteroscopy
*(Balloon camera inserted through the back passage* ○ Number ____

Flexible Sigmoidoscopy
*(Camera inserted through the back passage into the last part of the bowel only - usually only requires an enema)* ○ Number ____

A10. How were you referred for your most recent test?

I was referred directly by my GP (without seeing a hospital doctor) ○

The test was organised by a hospital doctor ○

I have regular tests to monitor a medical condition/ because of my family history ○

I was referred in another way (please tell us more in the box below) ○



Endosc Int Open | DOI 10.1055/a-2870-7457 | © The Author(s).

## Section B: Before coming to hospital for your test

In this section we want to find out about the time leading up to your test, before you came to hospital. Please answer all of the questions in this section by putting a '✔' next to the answer that applies to you.

| | | Strongly agree | Agree | Neither agree or disagree | Disagree | Strongly disagree |
|---|---|---|---|---|---|---|
| B1. | I was happy with the way I was referred for the test | ❍ | ❍ | ❍ | ❍ | ❍ |
| B2. | The time from first being referred to having the test done was satisfactory | ❍ | ❍ | ❍ | ❍ | ❍ |
| B3. | I felt able to change the appointment if it didn't suit me | ❍ | ❍ | ❍ | ❍ | ❍ |

B4. My appointment was cancelled or changed by the hospital

Yes ❍ No ❍ Not sure / can't remember ❍

| | | Strongly agree | Agree | Neither agree or disagree | Disagree | Strongly disagree |
|---|---|---|---|---|---|---|
| B5. | Before coming for the test, I was given enough information about what the test would involve | ❍ | ❍ | ❍ | ❍ | ❍ |
| B6. | After reading the information, I did not have any questions about the test | ❍ | ❍ | ❍ | ❍ | ❍ |



Endosc Int Open | DOI 10.1055/a-2870-7457 | © The Author(s).

## Before coming for the test:

| | Strongly agree | Agr ee | Neither agree or disagree | Disagr ee | Strongly disagree |
|---|---|---|---|---|---|
| B7. The instructions on what I needed to do before the test were easy to follow | ❍ | ❍ | ❍ | ❍ | ❍ |
| B8. I had enough time to discuss the test with the person who referred me | ❍ | ❍ | ❍ | ❍ | ❍ |
| B9. I felt anxious about what the test would involve | ❍ | ❍ | ❍ | ❍ | ❍ |
| B10. I was made anxious by talking to other people who had previously had the test | ❍ | ❍ | ❍ | ❍ | ❍ |

## Before coming for the test:

| | Strongly agree | Agr ee | Neither agree or disagree | Disagr ee | Strongly disagree |
|---|---|---|---|---|---|
| B11. I felt anxious about the results of the test | ❍ | ❍ | ❍ | ❍ | ❍ |
| B12. I expected to experience discomfort during the test | ❍ | ❍ | ❍ | ❍ | ❍ |
| B13. I expected to experience pain during the test | ❍ | ❍ | ❍ | ❍ | ❍ |
| B14. I was worried that inserting the tube/ camera would cause discomfort | ❍ | ❍ | ❍ | ❍ | ❍ |

## Section C: Preparing for your test

In this section we would like to know about your experience of preparing for the test. Please answer all of the questions in this section by putting a '✔' next to the answer that applies to you.

Please only complete this section if you have had a colonoscopy or retrograde enteroscopy.
If you had a different test please go to section D

| | | Strongly agree | Agree | Neither agree or disagree | Disagree | Strongly disagree |
|---|---|---|---|---|---|---|
| C1. | The bowel preparation had an unpleasant taste | ❍ | ❍ | ❍ | ❍ | ❍ |
| C2. | The bowel preparation tasted better than I had expected | ❍ | ❍ | ❍ | ❍ | ❍ |
| C3. | The volume *(amount)* of the bowel preparation was more than I had expected | ❍ | ❍ | ❍ | ❍ | ❍ |
| C4. | The amount of bowel preparation I had to drink was manageable | ❍ | ❍ | ❍ | ❍ | ❍ |
| C5. | I was worried that the bowel preparation would not clear my bowel properly | ❍ | ❍ | ❍ | ❍ | ❍ |
| C6. | I had enough privacy when getting ready for the test *(eg when changing clothes)* | ❍ | ❍ | ❍ | ❍ | ❍ |



Endosc Int Open | DOI 10.1055/a-2870-7457 | © The Author(s).

## Section D: At the hospital, before the test

In this section we would like to know about your experience of arriving at the hospital, getting ready and waiting for the test. Please complete ALL of the remaining sections of this survey, regardless of what test you had by putting a '✔' next to the answer that applies to you.

| | | Strongly agree | Agr ee | Neither agree or disagree | Disagr ee | Strongly disagree |
|---|---|---|---|---|---|---|
| D1. | The length of time I waited in the department was acceptable | ❍ | ❍ | ❍ | ❍ | ❍ |
| D2. | I was comfortable while sitting in the waiting area | ❍ | ❍ | ❍ | ❍ | ❍ |
| D3. | I felt able to ask the staff any questions before the test | ❍ | ❍ | ❍ | ❍ | ❍ |
| D4. | I had no unanswered questions before the test | ❍ | ❍ | ❍ | ❍ | ❍ |
| D5. | I had enough privacy when waiting for the test | ❍ | ❍ | ❍ | ❍ | ❍ |
| D6. | I had enough privacy when moving from the waiting area to the procedure room | ❍ | ❍ | ❍ | ❍ | ❍ |



Endosc Int Open | DOI 10.1055/a-2870-7457 | © The Author(s).

## Section E: During the test

In this section we would like to know about your experience of the test, from arriving in the procedure room until it was time to leave the procedure room. Please answer by putting a '✔' next to the answer that applies to you.

| | | Strongly agree | Agree | Neither agree or disagree | Disagree | Strongly disagree |
|---|---|---|---|---|---|---|
| E1. | During the test my dignity was maintained at all times | ❍ | ❍ | ❍ | ❍ | ❍ |
| E2. | I felt free to choose what medication to take *(eg sedative, no medication)* | ❍ | ❍ | ❍ | ❍ | ❍ |
| E3. | The medication worked as well as I had expected | ❍ | ❍ | ❍ | ❍ | ❍ |

*I did not have any medication* ❍

E4. I would have preferred the person doing the test *(inserting the tube or camera)* to be:

Male ❍ Female ❍ I have no preference ❍

E5. The person doing the test was:

Male ❍ Female ❍

| | | Strongly agree | Agree | Neither agree or disagree | Disagree | Strongly disagree |
|---|---|---|---|---|---|---|
| E6. | I felt confident that the person doing the test knew what they were doing | ❍ | ❍ | ❍ | ❍ | ❍ |
| E7. | The person doing the test did their best to put me at ease | ❍ | ❍ | ❍ | ❍ | ❍ |



Endosc Int Open | DOI 10.1055/a-2870-7457 | © The Author(s).

|  | Strongly agree | Agree | Neither agree or disagree | Disagree | Strongly disagree |
|---|---|---|---|---|---|
| E8. The other staff in the test room did their best to put me at ease | ❍ | ❍ | ❍ | ❍ | ❍ |
| E9. I was satisfied with the explanation given to me about the test | ❍ | ❍ | ❍ | ❍ | ❍ |
| E10. The person doing the test addressed any concerns I had | ❍ | ❍ | ❍ | ❍ | ❍ |
| E11. I felt I could stop the test if it became too uncomfortable | ❍ | ❍ | ❍ | ❍ | ❍ |
| E12. I felt embarrassed during the test | ❍ | ❍ | ❍ | ❍ | ❍ |
| E13. The test took longer than I expected | ❍ | ❍ | ❍ | ❍ | ❍ |

E14. How would you rate the level of discomfort you experienced during the test?

Please circle a number below:

| No discomfort | 0 | 1 | 2 | 3 | 4 | 5 | 6 | 7 | 8 | 9 | 10 | Worst discomfort imaginable |
|---|---|---|---|---|---|---|---|---|---|---|---|---|

E15. How long did the discomfort last during the test?

| I didn't have discomfort | A short time | A moderate time | A long time |
|---|---|---|---|
| ❍ | ❍ | ❍ | ❍ |

E16. How many times did you experience discomfort during the test?

| None | 1 or 2 times | 3 or 4 times | More than 4 | Constantly |
|---|---|---|---|---|
| ❍ | ❍ | ❍ | ❍ | ❍ |

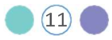
11

## Section E: During the test *continued...*

E17. How would you rate the level of pain you experienced during the test?

Please circle a number below:

| No pain | 0 | 1 | 2 | 3 | 4 | 5 | 6 | 7 | 8 | 9 | 10 | Worst pain imaginable |
|---|---|---|---|---|---|---|---|---|---|---|---|---|

E18. How long did the pain last during the test?

| I didn't have pain | A short time | A moderate time | A long time |
|---|---|---|---|
| ❍ | ❍ | ❍ | ❍ |

E19. How many times did you experience pain during the test?

| None | 1 or 2 times | 3 or 4 times | More than 4 times | Constantly |
|---|---|---|---|---|
| ❍ | ❍ | ❍ | ❍ | ❍ |

| | Strongly agree | Agr ee | Neither agree or disagree | Disagr ee | Strongly disagree |
|---|---|---|---|---|---|
| E20. Overall, I experienced more discomfort than I expected during the test | ❍ | ❍ | ❍ | ❍ | ❍ |
| E21. Overall, I experienced more pain than I expected during the test | ❍ | ❍ | ❍ | ❍ | ❍ |
| E22. I felt embarrassed by the discomfort I experienced | ❍ | ❍ | ❍ | ❍ | ❍ |
| E23. I felt embarrassed by the pain I experienced | ❍ | ❍ | ❍ | ❍ | ❍ |



Endosc Int Open | DOI 10.1055/a-2870-7457 | © The Author(s).

## Section F: After the test

In this section we would like to know about your experience after the test including the results, if you've had them. Please answer by putting a '✔' next to the answer that applies to you.

### After the test:

| | | Strongly agree | Agree | Neither agree or disagree | Disagree | Strongly disagree |
|---|---|---|---|---|---|---|
| F1. | I was satisfied by the explanation given to me by the person doing the test | ❍ | ❍ | ❍ | ❍ | ❍ |
| F2. | I had discomfort after the test | ❍ | ❍ | ❍ | ❍ | ❍ |
| F3. | It took longer than I expected to recover from the test | ❍ | ❍ | ❍ | ❍ | ❍ |
| F4. | I was worried about the test results | ❍ | ❍ | ❍ | ❍ | ❍ |

F5. Have you received the results of your test? *(Please tick all that apply)*

- Yes, I have received all of my test results ❍
- Yes, I have received some of my test results ❍
- No ❍



Endosc Int Open | DOI 10.1055/a-2870-7457 | © The Author(s).

## Section F: After the test *continued...*

| | | Strongly agree | Agree | Neither agree or disagree | Disagree | Strongly disagree |
|---|---|---|---|---|---|---|
| F6. | When I left the hospital, I was clear about what the next steps would be | ❍ | ❍ | ❍ | ❍ | ❍ |
| F7. | I was happy with the way I received the results of my test | ❍ | ❍ | ❍ | ❍ | ❍ |
| | *I do not have my results* ❍ | | | | | |
| F8. | I received the results of my test sooner than I had expected | ❍ | ❍ | ❍ | ❍ | ❍ |
| | *I do not have my results* ❍ | | | | | |



Endosc Int Open | DOI 10.1055/a-2870-7457 | © The Author(s).

## Section G: Overall experience

In this section we would like to know how you feel now about your overall experience. Please answer by putting a '✔' next to the answer that applies to you.

| After the test: | Strongly agree | Agree | Neither agree or disagree | Disagree | Strongly disagree |
|---|---|---|---|---|---|
| G1. Overall I was satisfied with my experience of the test | ❍ | ❍ | ❍ | ❍ | ❍ |

G2. If there is something else you would like to tell us about your test, please use the space below.

Thank you for taking the time to complete this survey



Endosc Int Open | DOI 10.1055/a-2870-7457 | © The Author(s).
